# 2,2′-[Ethyl­enebis(azanediyl­methyl­ene)]diphenol

**DOI:** 10.1107/S1600536809048831

**Published:** 2009-11-21

**Authors:** Ying-Ming Xu, Shan Gao, Seik Weng Ng

**Affiliations:** a College of Chemistry and Materials Science, Heilongjiang University, Harbin 150080, People’s Republic of China; b Department of Chemistry, University of Malaya, 50603 Kuala Lumpur, Malaysia

## Abstract

In the title compound, C_16_H_20_N_2_O_4_, the mol­ecule features a zigzag –CH_2_–NH–CH_2_–CH_2_–NH–CH_2_– chain whose ends are connected to the hydroxy­phenyl rings. The mol­ecules lies about a center of inversion. The imino group is a hydrogen-bond donor for the hydr­oxy group, which is a hydrogen-bond donor for the imino group of an adjacent mol­ecule. This latter inter­molecular hydrogen bonding leads to a layer structure.

## Related literature

The title compound was doubly-deprotonated, forming several tetra­dentate chelated metal complexes. For their crystal structures, see: Atwood *et al.* (1995 ▶, 1996 ▶); Borer *et al.* (1983 ▶); Bottcher *et al.* (1994 ▶); García-Zarracino *et al.* (2002 ▶); Henrick *et al.* (1984 ▶); Viswanathan *et al.* (1998 ▶); Xie *et al.* (2006 ▶); Yang *et al.* (2007 ▶).
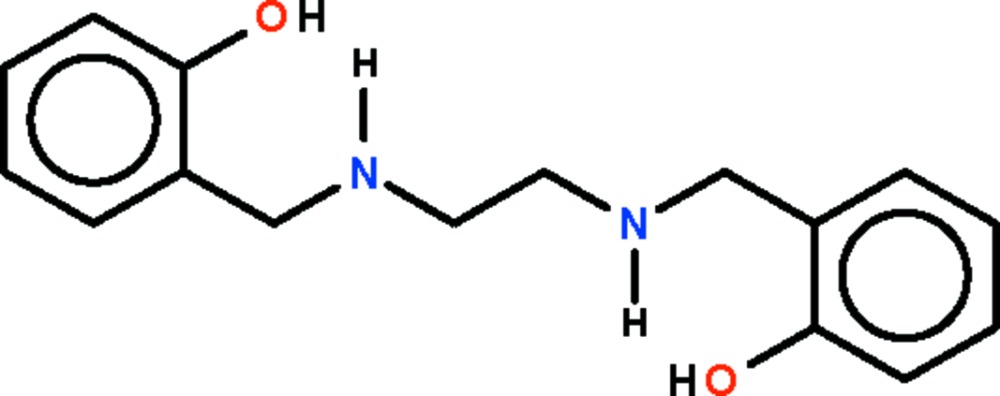


## Experimental

### 

#### Crystal data

C_16_H_20_N_2_O_2_
*M* *_r_* = 272.34
Monoclinic, 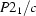

*a* = 15.263 (2) Å
*b* = 4.860 (1) Å
*c* = 9.770 (1) Å
β = 96.318 (3)°
*V* = 720.3 (2) Å^3^
*Z* = 2
Mo *K*α radiation
μ = 0.08 mm^−1^
*T* = 293 K
0.31 × 0.27 × 0.25 mm

#### Data collection

Rigaku R-AXIS RAPID IP diffractometer
Absorption correction: multi-scan (*ABSCOR*; Higashi, 1995 ▶) *T*_min_ = 0.975, *T*_max_ = 0.979
6726 measured reflections
1635 independent reflections
912 reflections with *I* > 2σ(*I*)
*R*_int_ = 0.055

#### Refinement

*R*[*F* ^2^ > 2σ(*F* ^2^)] = 0.052
*wR*(*F* ^2^) = 0.176
*S* = 1.09
1635 reflections
99 parameters
2 restraints
H atoms treated by a mixture of independent and constrained refinement
Δρ_max_ = 0.21 e Å^−3^
Δρ_min_ = −0.19 e Å^−3^

### 

Data collection: *RAPID-AUTO* (Rigaku, 1998 ▶); cell refinement: *RAPID-AUTO*; data reduction: *CrystalClear* (Rigaku/MSC, 2002 ▶); program(s) used to solve structure: *SHELXS97* (Sheldrick, 2008 ▶); program(s) used to refine structure: *SHELXL97* (Sheldrick, 2008 ▶); molecular graphics: *X-SEED* (Barbour, 2001 ▶); software used to prepare material for publication: *publCIF* (Westrip, 2009 ▶).

## Supplementary Material

Crystal structure: contains datablocks global, I. DOI: 10.1107/S1600536809048831/xu2679sup1.cif

Structure factors: contains datablocks I. DOI: 10.1107/S1600536809048831/xu2679Isup2.hkl

Additional supplementary materials:  crystallographic information; 3D view; checkCIF report

## Figures and Tables

**Table 1:** Hydrogen-bond geometry (Å, °)

*D*—H⋯*A*	*D*—H	H⋯*A*	*D*⋯*A*	*D*—H⋯*A*
O1—H1o⋯N1^i^	0.86 (1)	1.89 (1)	2.721 (2)	165 (3)
N1—H1n⋯O1	0.86 (1)	2.23 (2)	2.884 (2)	133 (2)

Symmetry code: (i) 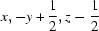.

**Fig. 1.**
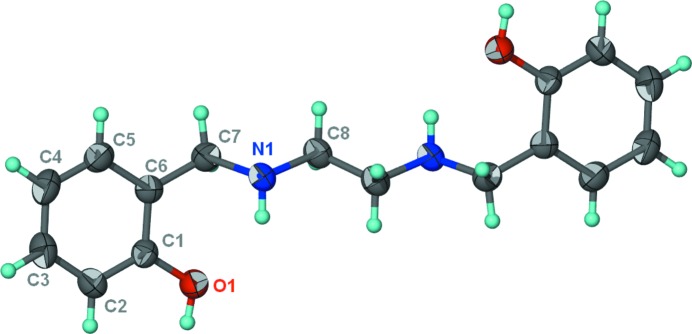
Thermal ellipsoid plot (Barbour, 2001) of the molecule of C16H20N2O2 at the 50% probability level; hydrogen atoms are drawn as spheres of arbitrary radius.

## supplementary crystallographic information

### Experimental

To a solution of salicylaldehyde (2.44 g, 20 mmol) in methanol was added a solution of ethylenediamine (0.6 ml, 10 mmol) in methanol. The solution was heated for two hours. The yellow Schiff base that was isolated upon evaporation of the solvent was reduced in absolute methanol by sodium borohydride. Colorless prismatic crystals were grown from a solution of the diamine in methanol.

### Refinement

Carbon-bound H-atoms generated geometrically (0.93–0.97 Å, U_iso_(H) = 1.2*U*_eq_(C)). The nitrogen- and oxygen-bound H-atoms were refined with a distance restraint of N–H = O–H = 0.85±0.01 Å; their temperature factors were refined.

### Crystal data

**Table:**

C_16_H_20_N_2_O_2_	*F*(000) = 292
*M**_r_* = 272.34	*D*_x_ = 1.256 Mg m^−^^3^
Monoclinic, *P*2_1_/*c*	Mo *K*α radiation, λ = 0.71073 Å
Hall symbol: -P 2ybc	Cell parameters from 3415 reflections
*a* = 15.263 (2) Å	θ = 4.0–27.4°
*b* = 4.860 (1) Å	µ = 0.08 mm^−^^1^
*c* = 9.770 (1) Å	*T* = 293 K
β = 96.318 (3)°	Prism, colorless
*V* = 720.3 (2) Å^3^	0.31 × 0.27 × 0.25 mm
*Z* = 2	

### Data collection

**Table:**

Rigaku R-AXIS RAPID IP diffractometer	1635 independent reflections
Radiation source: fine-focus sealed tube	912 reflections with *I* > 2σ(*I*)
graphite	*R*_int_ = 0.055
ω scan	θ_max_ = 27.4°, θ_min_ = 4.0°
Absorption correction: multi-scan (*ABSCOR*; Higashi, 1995)	*h* = −19→19
*T*_min_ = 0.975, *T*_max_ = 0.979	*k* = −6→6
6726 measured reflections	*l* = −12→11

### Refinement

**Table:**

Refinement on *F*^2^	Primary atom site location: structure-invariant direct methods
Least-squares matrix: full	Secondary atom site location: difference Fourier map
*R*[*F*^2^ > 2σ(*F*^2^)] = 0.052	Hydrogen site location: inferred from neighbouring sites
*wR*(*F*^2^) = 0.176	H atoms treated by a mixture of independent and constrained refinement
*S* = 1.09	*w* = 1/[σ^2^(*F*_o_^2^) + (0.0749*P*)^2^ + 0.1302*P*] where *P* = (*F*_o_^2^ + 2*F*_c_^2^)/3
1635 reflections	(Δ/σ)_max_ = 0.001
99 parameters	Δρ_max_ = 0.21 e Å^−^^3^
2 restraints	Δρ_min_ = −0.19 e Å^−^^3^

### Fractional atomic coordinates and isotropic or equivalent isotropic displacement parameters (Å^2^)

**Table:**

	*x*	*y*	*z*	*U*_iso_*/*U*_eq_	
O1	0.34270 (10)	0.3118 (4)	0.13029 (16)	0.0555 (5)	
N1	0.38397 (11)	0.4899 (4)	0.41165 (18)	0.0451 (5)	
C1	0.25836 (14)	0.3821 (5)	0.1507 (2)	0.0439 (6)	
C2	0.18572 (15)	0.2708 (5)	0.0744 (2)	0.0552 (6)	
H2A	0.1932	0.1404	0.0070	0.066*	
C3	0.10190 (15)	0.3510 (6)	0.0972 (3)	0.0622 (7)	
H3	0.0532	0.2732	0.0460	0.075*	
C4	0.09036 (16)	0.5466 (6)	0.1958 (3)	0.0624 (7)	
H4	0.0340	0.6040	0.2103	0.075*	
C5	0.16325 (16)	0.6564 (5)	0.2727 (2)	0.0564 (7)	
H5	0.1552	0.7871	0.3397	0.068*	
C6	0.24808 (13)	0.5773 (5)	0.2530 (2)	0.0443 (6)	
C7	0.32748 (15)	0.6983 (5)	0.3364 (2)	0.0524 (6)	
H7A	0.3621	0.7987	0.2754	0.063*	
H7B	0.3079	0.8285	0.4019	0.063*	
C8	0.46843 (13)	0.6100 (5)	0.4701 (2)	0.0497 (6)	
H8A	0.4579	0.7403	0.5418	0.060*	
H8B	0.4947	0.7091	0.3988	0.060*	
H1O	0.3458 (18)	0.209 (5)	0.0600 (19)	0.081 (10)*	
H1N	0.3943 (15)	0.370 (4)	0.3504 (19)	0.060 (7)*	

### Atomic displacement parameters (Å^2^)

**Table:**

	*U*^11^	*U*^22^	*U*^33^	*U*^12^	*U*^13^	*U*^23^
O1	0.0458 (10)	0.0730 (13)	0.0477 (10)	0.0062 (8)	0.0060 (7)	−0.0106 (8)
N1	0.0441 (10)	0.0469 (12)	0.0432 (10)	−0.0043 (8)	0.0005 (8)	−0.0013 (9)
C1	0.0434 (12)	0.0475 (13)	0.0409 (11)	0.0033 (10)	0.0055 (9)	0.0053 (10)
C2	0.0546 (14)	0.0616 (16)	0.0482 (13)	−0.0008 (11)	−0.0005 (11)	−0.0064 (12)
C3	0.0457 (14)	0.0746 (19)	0.0642 (16)	−0.0037 (12)	−0.0036 (12)	0.0023 (14)
C4	0.0465 (13)	0.0746 (19)	0.0660 (16)	0.0126 (13)	0.0065 (12)	0.0082 (14)
C5	0.0565 (14)	0.0592 (16)	0.0536 (14)	0.0127 (12)	0.0074 (11)	0.0000 (11)
C6	0.0489 (13)	0.0424 (13)	0.0418 (11)	0.0012 (9)	0.0050 (10)	0.0039 (9)
C7	0.0570 (14)	0.0458 (14)	0.0532 (13)	0.0055 (11)	0.0014 (11)	0.0000 (11)
C8	0.0480 (13)	0.0493 (15)	0.0510 (13)	−0.0079 (10)	0.0009 (10)	−0.0009 (11)

### Geometric parameters (Å, °)

**Table:**

O1—C1	1.367 (2)	C4—C5	1.380 (4)
O1—H1O	0.86 (1)	C4—H4	0.9300
N1—C8	1.472 (3)	C5—C6	1.384 (3)
N1—C7	1.473 (3)	C5—H5	0.9300
N1—H1N	0.86 (1)	C6—C7	1.504 (3)
C1—C2	1.377 (3)	C7—H7A	0.9700
C1—C6	1.399 (3)	C7—H7B	0.9700
C2—C3	1.379 (3)	C8—C8^i^	1.513 (4)
C2—H2A	0.9300	C8—H8A	0.9700
C3—C4	1.379 (4)	C8—H8B	0.9700
C3—H3	0.9300		
			
C1—O1—H1O	113.5 (19)	C4—C5—H5	119.1
C8—N1—C7	111.12 (17)	C6—C5—H5	119.1
C8—N1—H1N	108.6 (16)	C5—C6—C1	117.9 (2)
C7—N1—H1N	105.1 (16)	C5—C6—C7	121.7 (2)
O1—C1—C2	122.6 (2)	C1—C6—C7	120.34 (19)
O1—C1—C6	117.04 (19)	N1—C7—C6	113.21 (18)
C2—C1—C6	120.4 (2)	N1—C7—H7A	108.9
C1—C2—C3	120.5 (2)	C6—C7—H7A	108.9
C1—C2—H2A	119.7	N1—C7—H7B	108.9
C3—C2—H2A	119.7	C6—C7—H7B	108.9
C2—C3—C4	120.0 (2)	H7A—C7—H7B	107.8
C2—C3—H3	120.0	N1—C8—C8^i^	111.3 (2)
C4—C3—H3	120.0	N1—C8—H8A	109.4
C3—C4—C5	119.4 (2)	C8^i^—C8—H8A	109.4
C3—C4—H4	120.3	N1—C8—H8B	109.4
C5—C4—H4	120.3	C8^i^—C8—H8B	109.4
C4—C5—C6	121.8 (2)	H8A—C8—H8B	108.0
			
O1—C1—C2—C3	−179.0 (2)	C2—C1—C6—C5	−1.0 (3)
C6—C1—C2—C3	0.4 (4)	O1—C1—C6—C7	−0.7 (3)
C1—C2—C3—C4	0.8 (4)	C2—C1—C6—C7	179.9 (2)
C2—C3—C4—C5	−1.2 (4)	C8—N1—C7—C6	169.35 (18)
C3—C4—C5—C6	0.6 (4)	C5—C6—C7—N1	122.4 (2)
C4—C5—C6—C1	0.6 (4)	C1—C6—C7—N1	−58.5 (3)
C4—C5—C6—C7	179.7 (2)	C7—N1—C8—C8^i^	−171.9 (2)
O1—C1—C6—C5	178.39 (19)		

Symmetry codes: (i) −*x*+1, −*y*+1, −*z*+1.

### Hydrogen-bond geometry (Å, °)

**Table:**

*D*—H···*A*	*D*—H	H···*A*	*D*···*A*	*D*—H···*A*
O1—H1o···N1^ii^	0.86 (1)	1.89 (1)	2.721 (2)	165 (3)
N1—H1n···O1	0.86 (1)	2.23 (2)	2.884 (2)	133 (2)

Symmetry codes: (ii) *x*, −*y*+1/2, *z*−1/2.
